# The Redemption of Noise: Inference with Neural Populations

**DOI:** 10.1016/j.tins.2018.09.003

**Published:** 2018-11

**Authors:** Rodrigo Echeveste, Máté Lengyel

**Affiliations:** 1 Computational and Biological Learning Laboratory, Department of Engineering, University of Cambridge, Cambridge, UK; 2 Department of Cognitive Science, Central European University, Budapest, Hungary

**Keywords:** Bayesian inference, neural network, neural variability, perception, cortex, uncertainty

## Abstract

In 2006, Ma *et al*. presented an elegant theory for how populations of neurons might represent uncertainty to perform Bayesian inference. Critically, according to this theory, neural variability is no longer a nuisance, but rather a vital part of how the brain encodes probability distributions and performs computations with them.

The brain faces a daunting task and solves it with such ease that we are rarely even aware of it: making sense of the outside world based on a set of noisy and incomplete sensory inputs. Our visual system, for example, needs to deal with partially occluded objects, or infer 3D shapes from 2D images in our retinas, all the while relying on intrinsically noisy photoreceptor activations. The Bayesian theory of probabilistic inference provides an optimal solution for dealing with the uncertainty that is inherent in sensory processing, but which classical theories of sensory processing typically eschew. The key is to represent uncertainty in the form of probability distributions, such that instead of just computing a single best estimate of a stimulus feature, a posterior distribution over that feature is computed, quantifying the strength of the observer’s ‘belief’ that the stimulus may take on any particular value given the evidence provided by our senses.

A probabilistically appropriate representation of uncertainty is indispensable for the brain in at least three contexts: first, when fusing information from multiple information sources (e.g., sensory modalities, or memory), each of which may be unreliable on its own; second, when making decisions that require combining incomplete sensory information with subjective utilities; and finally, for updating its internal models of the world over time, so that it remains well calibrated [1]. Indeed, behavioral studies of perception (and other cognitive functions) had long indicated that the brain must somehow represent uncertainty, as underscored by the observation that it can sometimes perform near the Bayesian optimum [2]. A critical question is then: how are probability distributions encoded in the responses of neural populations?

The seminal paper of Wei Ji Ma, Jeffrey Beck, Peter Latham, and Alexandre Pouget [3] proposed a solution to this question in the form of probabilistic population codes (PPCs). Similar schemes, according to which populations of neurons could encode probability distributions about a stimulus, had earlier been studied by Pouget and others [4]. Among the key novelty points here was a biologically plausible implementation that would allow neural circuits to encode and operate with probability distributions. Critically, this approach relied on neural activities being variable or noisy, therefore marking a departure from the traditional view of variability in the brain being a nuisance to that of variability being an essential part of performing probabilistic inference.

The starting point for Ma *et al*. [3] was the well-known experimental observation that the same stimulus repeatedly presented to an observer will produce each time a different pattern of activation in cortical neurons that are tuned to specific features of that stimulus (Figure 1, encoding). Conversely, a given pattern of activity in the brain could arise in response to several possible stimuli. This probabilistic relationship between stimuli and responses can be formalized by P(response | stimulus), expressing the probability of obtaining a particular population response given a stimulus. The same quantity, P(response | stimulus), also expresses how likely, given a particular neural response, a stimulus value is (Figure 1, decoding). This likelihood function is central for computing the Bayesian posterior distribution over the stimulus (via Bayes’ rule), and it represents uncertainty in a fairly straightforward manner. If the population response is such that the likelihood is narrowly peaked around a single stimulus, there is little uncertainty; conversely, if the likelihood is a broad function of the stimulus, then it expresses a high level of uncertainty. While classical approaches to neural coding tend to treat the fact that the stimulus cannot be identified unequivocally from the response as a nuisance, PPCs thrive on this ambiguity: according to the theory of PPCs, each population response inherently encodes uncertainty over stimuli, just as required for performing proper Bayesian inference. The critical step then was to show that the way circuit dynamics transform one particular population response into another corresponds to a probabilistically meaningful transformation of one likelihood (represented by the first response) to another one (represented by the second response).

**Figure 1:**
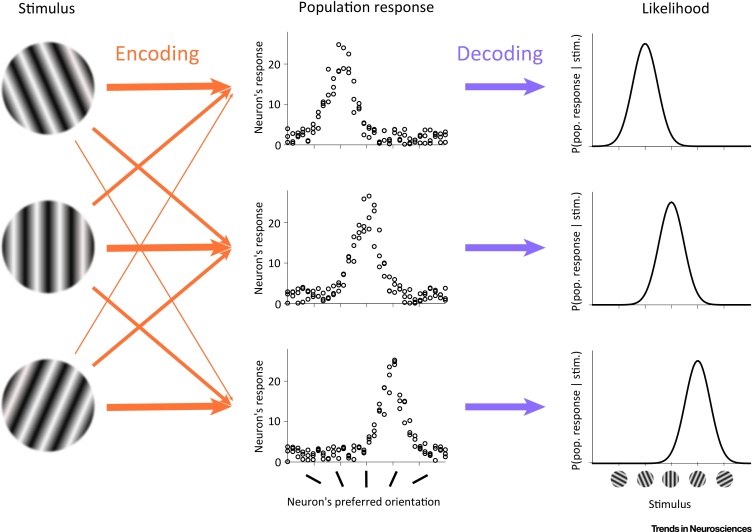
Probabilistic Encoding and Decoding of Stimuli in Population Responses. Encoding (orange arrows): the mapping from stimuli to responses. On each trial, the same stimulus (left) evokes a different pattern of neural responses in a population of tuned neurons (middle), such that some responses occur with a higher probability than others (arrows emanating from the same stimulus, arrow width represents response probability). For different stimuli, these response probabilities will typically be different (compare arrows pointing to the same response, emanating from different stimuli). Decoding (purple arrows): the mapping from responses to stimuli. Given the inherently probabilistic nature of encoding, the same neural response pattern could have originated from several possible stimuli. The likelihood of the stimulus quantifies the probability with which any given value of the stimulus might have evoked the actual population responses, P(response | stimulus) (right).

A paradigmatic transformation of likelihoods arises in situations when different sensory cues convey information about the value of a stimulus that needs to be inferred. Examples include visual and auditory cues reporting about the location of an object [3] (Figure 2A), or sequentially received packets of sensory information about the underlying direction of motion in an evidence accumulation task using a random dot kinematogram [5]. In these situations, each cue gives rise to a different population response (Figure 2B, blue and green) and thus a different likelihood function (Figure 2C, blue and green), but the brain ultimately needs to compute the likelihood of the stimulus combining all the information conveyed by the cues. The probabilistically correct way to combine the individual likelihoods (as long as they represent independent pieces of information) is to multiply them; the likelihood of a stimulus value considering all population responses should simply be the product of the individual likelihoods of this stimulus value associated with each response (Figure 2C, red).

**Figure 2:**
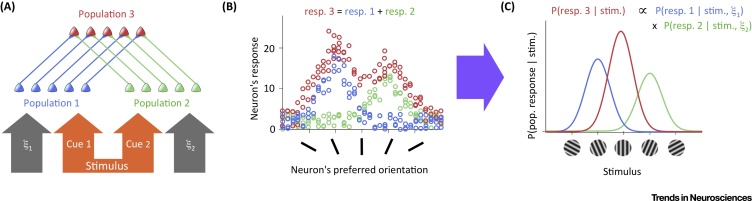
Cue Combination by PPCs. The product of likelihoods is computed by summing neural responses. (A) Two cues, each encoded (orange arrows) by the stochastic responses of a neural population (blue and green), convey information about the same underlying stimulus. These two populations provide feed-forward input to the output layer (red). Responses in each input population may also depend on nuisance parameters (ξ_1_ and ξ_2_, gray arrows). (B) Example neural responses in the three populations (cf. Figure 1, middle). Responses in the output population are the (weighted) sum of the responses in the input populations (top, weighting factors are omitted for clarity). (C) The likelihood functions that can be decoded (purple arrow) from the responses of each of the three populations (cf. Figure 1, right). While the input layers only encode the likelihood of the stimulus given the information available in their respective cues (blue and green), the output layer represents the combined likelihood of the stimulus given all available information (red); that is, the product of individual input likelihoods (top). Note that the likelihood encoded by the output layer can be interpreted without knowledge of the nuisance parameters.

A key contribution of Ma *et al*. [3] was to show that, under biologically plausible conditions, this combined likelihood can be represented by a strikingly simple transformation of the population responses associated with the individual cues: their sum (Figure 2B, red). In other words, a two-layer feed-forward neural network in which neurons in the output layer take an appropriately weighted sum of the neural responses in the input populations performs optimal cue combination, computing the product of input likelihood functions. Analogously, for evidence accumulation, the output layer needs to compute a cumulative sum over time of the responses in the input layer [5], just as in the much celebrated drift-diffusion model of decision making. Moreover, although the mathematical form of the decoding function that maps from neural responses to the likelihood (Figure 1, purple arrow) can in general be arbitrarily complex, in PPCs it admits a particularly simple form: both the individual input likelihoods represented in the input layer and the combined likelihood represented in the output layer can be decoded by computing a linear function (an appropriately weighted sum) of the corresponding neural responses. Linear decoding has a long history in systems neuroscience, viewed by many as the kind of representation the brain strives to achieve [6], and something that cortical neurons may easily implement [7]. Intriguingly, it is precisely this linear decodability of likelihoods from the responses of the input layer that by itself guarantees both that the summation of these responses by the network implements optimal cue combination and that the resulting sum is also linearly decodable.

One complication, which Ma *et al*. [3] noted as well, is that responses in a given neural population typically depend on many other sensory features (e.g., image contrast), or even stimulus-independent factors (e.g., attention), beside the particular property of the stimulus that a brain area may be inferring (e.g., the orientation of a line segment in the primary visual cortex). The problem is that, in general, in the presence of these nuisance parameters, the likelihood of the stimulus would only be linearly decodable if the values of all the nuisance parameters were already known with certainty to the decoder – a clearly untenable assumption. Thus, Ma *et al*. [3] went on to show that as long as neural responses satisfy two additional conditions, linear decodability of the stimulus likelihood will be preserved even in the presence of nuisance parameters. First, the distribution of responses should be Poisson-like: nuisance parameters should scale together the mean and the (co)variance of responses, such that the ratio of the mean and variance (the Fano factor) remains constant. This seems consistent with the often-observed (or at least assumed) property of cortical spike trains: that they resemble a Poisson process (i.e., they have a Fano factor that remains approximately constant [8], though see [9]). Second, the tuning curves (and noise covariance) of neurons should be translation-invariant, which effectively means that the population should always express roughly the same kind of response pattern, which is simply shifted as the stimulus is changed (as in Figure 1). Translation invariance has also been a standard assumption in theoretical studies of population codes [8] even if it is probably a rather crude approximation of reality [10].

The lasting impact of Ma *et al*. [3] is evident in how it motivated specific experimental tests and led to new theoretical developments in the study of probabilistic computations. Some of the detailed assumptions (or, conversely, predictions) that the PPC theory makes about neural responses may be difficult to test directly, or may even be inaccurate. For example, Fano factors and even the detailed patterns of response covariances may change with stimulus onset, image contrast, and other parameters or task events 11, 12, thus violating the Poisson-like assumption of PPCs. The strictly deterministic processing (summation of input responses) in the output layer of the PPC architecture, in contrast to the intrinsically stochastic activity assumed in its input layer, may also be hard to reconcile with what we know about the operation of cortical circuits. Nevertheless, as we saw, for PPCs the critical question is whether the stimulus is linearly decodable from neural responses, and whether it remains so even in the presence of nuisance parameters. This prediction has been confirmed experimentally [13]. One potential caveat is that the experimental tests so far have been conducted with at most one nuisance parameter (e.g., image contrast), while theoretical studies suggest that a more diverse (and probably more realistic) set of nuisance parameters (such as phase, aperture, or even object identity) can easily abolish linear decodability and make the resulting population code different from a PPC [12]. Indeed, there have been advances in exploring how PPCs might deal with nuisance parameters in more sophisticated ways [14]. In addition, fundamentally different proposals have been put forth for how variability in neural responses may support probabilistic inference without requiring linear decodability 12, 15. Continuing the journey started by Ma *et al*. [3], these theories are leading to specific, distinct, and experimentally testable predictions that will advance our understanding of the neural bases of probabilistic inference, and more broadly, of how our brains make sense of the surrounding world.
